# Reciprocal regulation of DGCR5 and miR-320a affects the cellular malignant phenotype and 5-FU response in pancreatic ductal adenocarcinoma

**DOI:** 10.18632/oncotarget.18377

**Published:** 2017-06-06

**Authors:** Sun Yong, Yu Yabin, Zhou Bing, Zhu Chuanrong, Gu Dianhua, Zhang Jianhuai, Yuan Weidong, Wang Shuming, Liu Ling

**Affiliations:** ^1^ Department of Hepatobiliary and Pancreatic Surgery, Huai’an First People’s Hospital, Nanjing Medical University, Huai'an, Jiangsu 223300, People’s Republic of China

**Keywords:** PDAC, DGCR5, miR-320a, lncRNA

## Abstract

Pancreatic ductal adenocarcinoma (PDAC) is one of the most aggressive and lethal malignancies. Long non-coding microRNAs (lncRNAs) are a newly discovered type of regulatory molecule with both diagnostic and prognostic value, but the role of lncRNA in PDAC has not been well investigated until now. Here, we present evidence that shows that the lncRNA DGCR5 is significantly reduced in PDAC tissues as well as in PDAC cell lines and that the downregulation of DGCR5 predicts poor prognosis. Ectopic expression of DGCR5 inhibits the proliferation and migration, and promotes 5-FU resistances of PDAC cells. Further experiments demonstrated that DGCR5 and miR-320a regulate each other in a reciprocal manner and that DGCR5 reverses the inhibition of PDCD4 by miR-320a, which is involved in the regulation of the PDAC cell phenotype and response to 5-FU. Our findings provide novel information about the functions of lncRNAs in PDAC, some of which might be beneficial to the precise diagnosis, prognosis and individualized therapy of patients with PDAC in the future.

## INTRODUCTION

Pancreatic cancer (PC) is one of the most common lethal malignant diseases worldwide, as PC has almost equal mortality and incidence rates [1] and is ranked as the fourth and seventh leading cause of cancer-related deaths in the USA and China, respectively [2, 3]. Among the various types of pancreatic cancer, pancreatic ductal adenocarcinoma (PDAC) is the most aggressive and lethal, and it accounts for approximately 85% of all pancreatic cancers. Surgical resection combined with the appropriate chemotherapeutic regimen remains the most effective strategy for PDAC, but only a small fraction of patients are eligible for surgical resection due to late diagnosis of the disease [4, 5]. To diagnose the disease early and to increase the tumor-free survival rate of patients with PDAC, it is critical to explore the detailed molecular mechanism that underlies PDAC tumorigenesis and to search for new molecular targets that are involved in the growth and metastasis of PDAC. These targets may be used as diagnostic factors and/or therapeutic targets in PDAC.

In recent decades, the role of non-coding RNA in the development of human diseases, including cancers, has been widely studied. Although microRNA has been well studied, the investigation into long non-coding RNA has just begun. LncRNAs, which belong to a class of ncRNA >200 nucleotides in length, have been implicated in various biological processes such as chromatin reprogramming, cis- or trans- regulation of neighboring genes and post-transcriptional regulation of mRNA processing [6–8]. LncRNAs have also been demonstrated to function sponges that regulate the levels as well as the activities of microRNAs [9, 10]. LncRNAs can also act as competing endogenous RNAs (ceRNAs), which compete with protein-coding genes for microRNA binding; this reverses the inhibition of protein-coding genes [11–13]. For instance, H19, HOTAIR (HOX transcript antisense intergenic RNA), HOTTIP (HOXA distal transcript antisense RNA), MALAT1 (metastasis-associated lung adenocarcinoma transcript 1), and PVT1 (plasmacytoma variant translocation 1) have been demonstrated to be associated with PC, but the detailed mechanisms are not well understood [5]. DiGeorge syndrome critical region gene 5 (DGCR5), which is also known as Linc0037, was first reported to be downregulated in Huntington’s disease but has also been reported to be downregulated in PDAC [14]. Our previous studies also indicated the decreased expression of DGCR5 in PDAC, but the role of DGCR5 in the development of PDAC is still unclear.

In this study, we investigated the possible role of DGCR5 in PDAC cells and further explored its possible mechanism. We found that DGCR5 is significantly reduced in clinical PDAC samples and PDAC cell lines and that a downregulated DGCR5 level is associated with tumor-free survival and the malignant phenotype of PDAC cells. In addition, we found that DGCR5 and miR-320a mutually regulate each other and that DGCR5 reverses the inhibition of PDCD4 by miR-320. Our study elucidates how DGCR5 affects the proliferation, migration and the resistance of PDAC cells to 5-FU, and provides a theoretical basis for the diagnosis and treatment of PDAC.

## RESULTS

### Downregulation of the lncRNA DGCR5 in PDAC and its clinical significance

**Figure 1:**
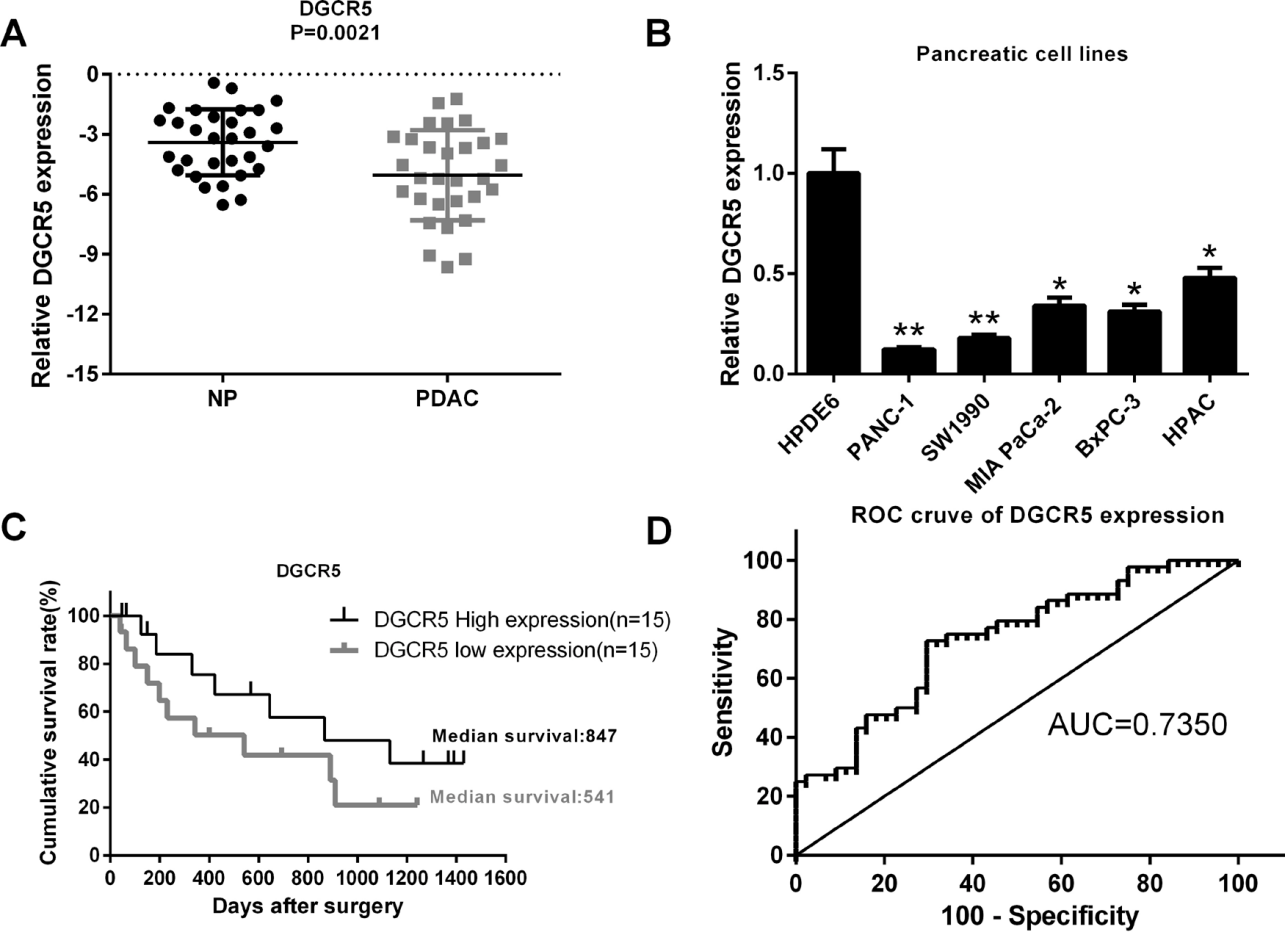
The lncRNA DGCR5 is downregulated in PDAC and is associated with the clinical outcome of patients with PDAC **(A)** The expression of the lncRNA DGCR5 in thirty paired clinical PDAC tumor tissues and adjacent non-tumor tissues was measured by real-time PCR, and the expression level of DGCR5 was normalized to that of GAPDH. **(B)** The expression level of DGCR5 in several PDAC cell lines and in the non-PDAC pancreatic cell line HPDE6 was measured by real-time PCR; GAPDH served as a control. The DGCR5 level in HPDE6 was set to one. **(C)** The survival curve of PDAC patients with either a high or a low DGCR5 expression level was generated, and the median survival period was indicated in the curve. **(D)** The ROC curve of DGCR5 for prediction of PDAC was made, and the area under the curve was indicated. *p<0.05; **p<0.01.

In our preliminary study, we found that several lncRNAs were differentially expressed in clinical PDAC samples compared with normal pancreatic tissues according to microarray experiments (data not shown). We also found that DGCR5 was significantly downregulated in PDAC. In addition, by deep sequencing, DGCR5 was demonstrated to be under-expressed [15]. To further confirm the downregulation of DGCR5 in PDAC, we determined the expression level of DGCR5 in thirty pairs of clinical PDAC samples and in matched adjacent non-tumorous pancreatic tissues. We found that the expression of DGCR5 in PDAC was significantly lower than that of non-tumorous pancreatic tissues (Figure 1A). In accordance with the result in clinical samples, the expression level of DGCR5 in several PDAC cell lines was lower compared with that in non-malignant HPDE6 cells (Figure 1B). These data imply that DGCR5 may be a tumor suppressor gene in PDAC. To obtain further insight, we divided the clinical PDAC samples into two groups according to the expression of DGCR5 and compared the survival curve of patients in these groups. We found that the group with higher DGCR5 expression had a longer median survival time than the group with lower DGCR5 expression (847 days VS. 541 days, Figure 1C), which further supports the tumor suppressive role of DGCR5 in PDAC. We then performed a receiver operating characteristic curve analysis to evaluate the diagnostic value of DGCR5 in PDAC. The area under the curve (AUC) was 0.735 (Figure 1D). Together, the results above demonstrate that DGCR5 is downregulated in PDAC and might be used as a predictive factor for PDAC.

### DGCR5 inhibits the malignant phenotype of PDAC cells

**Figure 2:**
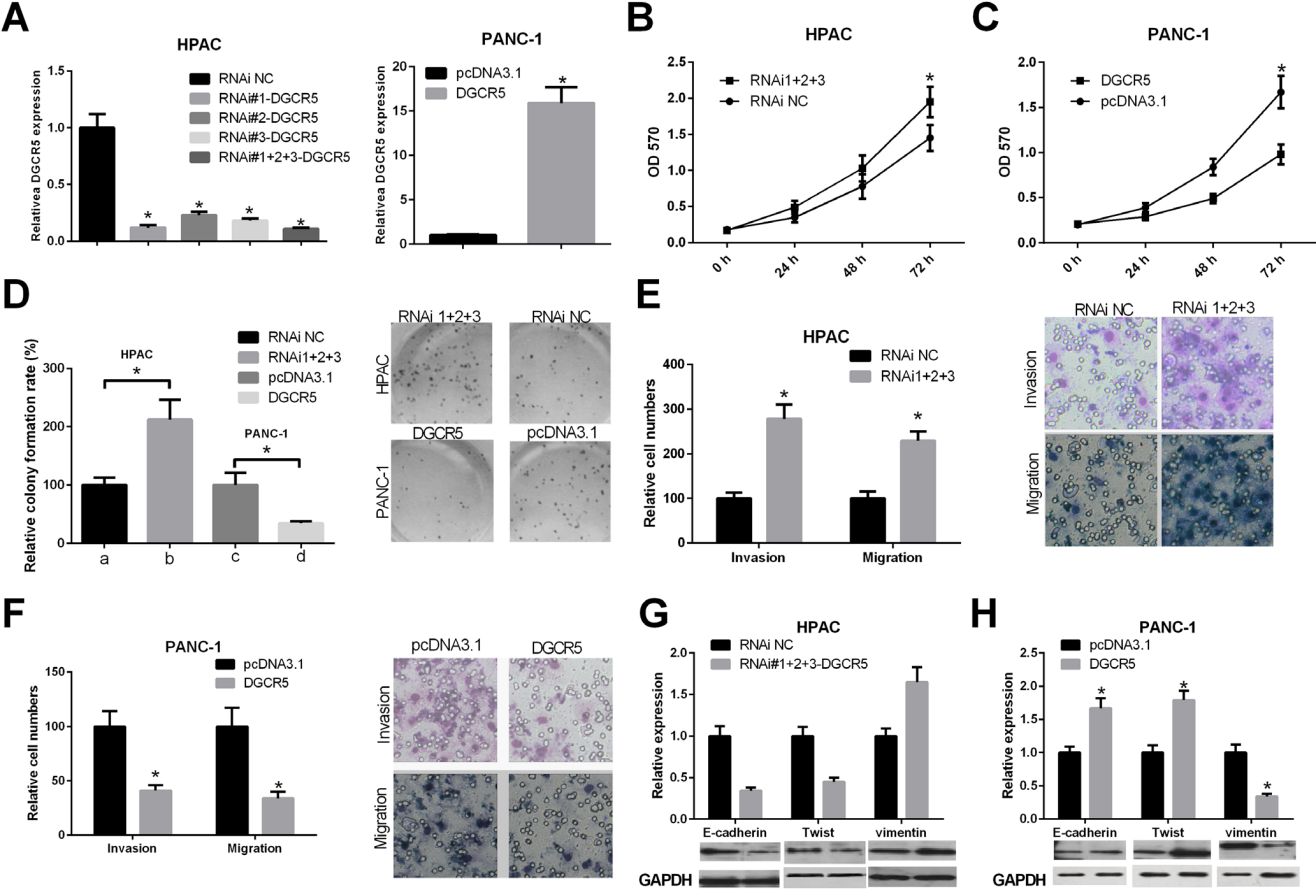
The role of lncRNA DGCR5 in the malignant phenotype of PDAC cells **(A)** The efficiency of the siRNA against DGCR5 and the DGCR5 over-expression plasmid was validated in HPAC and PANC-1 cells, respectively. The level of DGCR5 was quantified by qRT-PCR, and the level of GAPDH mRNA served as a control. **(B)** HPAC cells were transfected with the pool of siRNAs against DGCR5 or the control siRNA, and the proliferation of the cells was measured by CCK-8 assay. **(C)** PANC-1 cells were transfected with the DGCR5 overexpression plasmid or the control plasmid, and the proliferation of the cells was measured by CCK-8 assay. **(D)** HPAC and PANC-1 cells were transfected as described above, and a colony formation assay was performed. The histogram represents data from three independent experiments, and representative images of colony formation are also shown. **(E)** HPAC cells were transfected as described above and a Transwell migration and invasion assay was performed. The histogram represents data from three independent experiments, and representative images of migration and invasion are also shown. **(F)** PANC-1 cells were transfected as described above, and a Transwell migration and invasion assay was performed. The histogram represents data from three independent experiments, and representative images of migration and invasion are also shown. (**G** and **H**) HPAC and PANC-1 cells were transfected as described above, and the protein levels of E-cadherin, Twist and Vimentin were measured by western blot; GAPDH protein served as a loading control. The histogram represents data from three independent experiments. *p<0.05.

To study the role of DGCR5 in the regulation of the phenotype of PDAC cells, we first synthesized siRNA for DGCR5 and constructed a DGCR5 overexpression plasmid. We validated the efficiency of si-DGCR5 in HAPC cells, which have a relatively high expression level of DGCR5. The efficiency of the DGCR5 overexpression plasmid was validated in PANC-1 cells, which have a relatively low expression level of DGCR5 (Figure 2A). Then, a CCK-8 assay was performed to determine the role of DGCR5 in the proliferation of PDAC cells. The knockdown of DGCR5 in HAPC cells promoted their proliferation (Figure 2B), while overexpression of DGCR5 in PANC-1 cells inhibited their proliferation (Figure 2C). We then conducted a colony formation assay to evaluate the impact of DGCR5 on the colony formation ability of PDAC cells. The results showed that the knockdown of endogenous DGCR5 increased the colony formation ability of HPAC cells, while exogenous DGCR5 reduced the colony formation ability of PANC-1 cells (Figure 2D). Since metastasis is the component of cancer that causes death, we investigated the role of DGCR5 in the migration and invasiveness of PDAC cells by a Transwell migration and invasion assay. Si-DGCR5 significantly increased the migration and invasion abilities of HAPC cells (Figure 4E), while over-expression of DGCR5 significantly reduced the migration and invasion abilities of PANC-1 cells. EMT is activated in many cancer types and is regulated in many ways. We found that DGCR5 could increase the expression of the epithelial makers E-cadherin and Twist and could reduce the expression of the mesenchymal maker Vimentin (Figure 2G and 2H). Taken together, the above results demonstrate that DGCR5 inhibits the malignant phenotype of PDAC cells.

**Figure 3:**
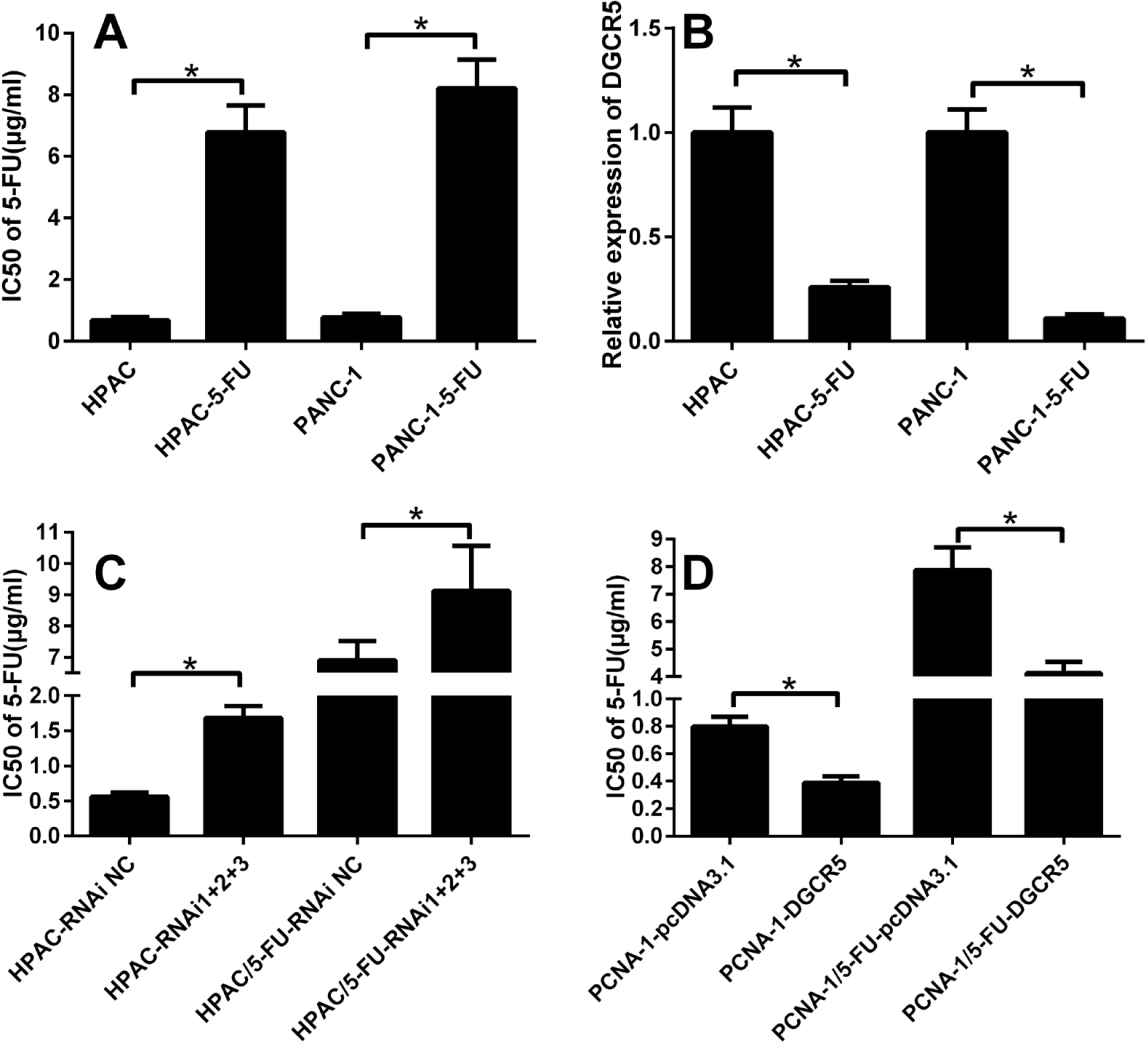
DGCR5 is negatively correlated with the response of PDAC cells to 5-FU **(A)** The IC50 of 5-FU in parental HPAC, PANC-1 cells and in the respective 5-FU- resistant cells was measured by MTT assay, and the histogram represents data from three independent experiments. **(B)** The expression level of DGCR5 in parental and 5-FU-resistant HPAC and PANC-1 cells was measured by qRT-PCR; the level of GAPDH mRNA served as a control, and the level of DGCR5 in parental cells was set to one. **(C)** The IC50 of 5-FU in parental or 5-FU-resistant HPAC cells transfected with the DGCR5 siRNA pool or the control siRNA was measured by MTT assay, and the histogram represents data from three independent experiments. **(D)** The IC50 of 5-FU in parental or 5-FU-resistant PANC-1 cells transfected with the DGCR5 expression plasmid or the control plasmid was measured by MTT assay, and the histogram represents data from three independent experiments. *p<0.05.

**Figure 4:**
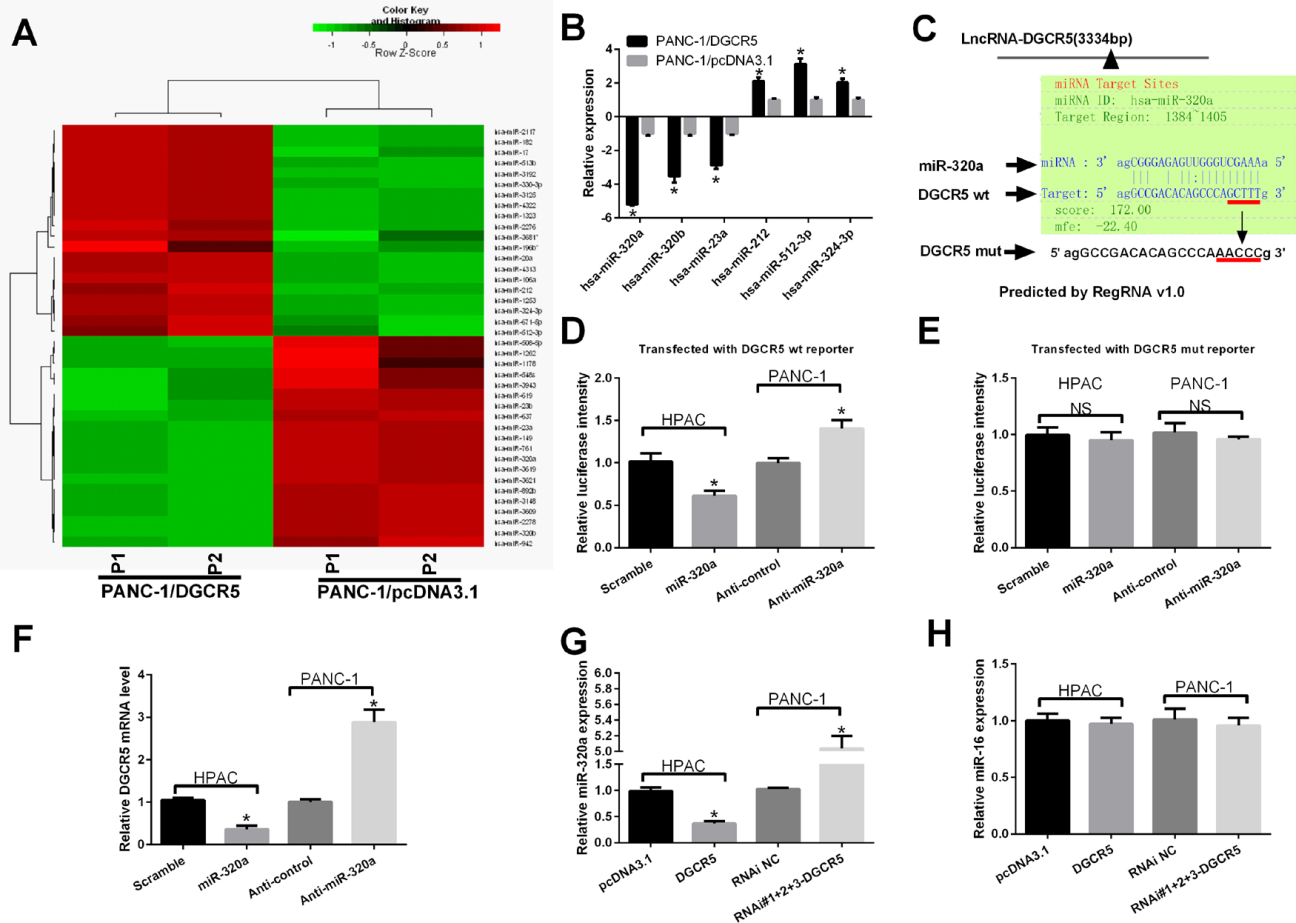
Co-regulation of DGCR5 and miR-320a in PDAC cells **(A)** Heat map of differentially expressed miRNAs in PANC-1 cells transfected with the DGCR5 expression plasmid or the control plasmid. **(B)** The discrepant expression of miRNAs in A was confirmed by qRT-PCR, and GAPDH mRNA served as a control. **(C)** The target site for miR-320a on DGCR5 was predicted by RegRNA v1.0. (**D** and **E**) A luciferase reporter plasmid containing the wild type **(D)** or mutant **(E)** miR-320a targeting site on DGCR5 was transfected simultaneously with miR-320a or anti-miR-320a into HAPC cells or PANC-1 cells, respectively. Then, 24 hours post-transfection the luciferase activity was measured; the activity of firefly luciferase was normalized to that of Renilla luciferase. **(F)** HPAC and PANC-1 cells were transfected with miR-320 mimics or with anti-miR-320, respectively. The RNA level of DGCR5 was measured by qRT-PCR, and GAPDH mRNA served as a control. **(G)** HPAC and PANC-1 cells were transfected with DGCR5 or si-DGCR5, respectively. The RNA level of miR-320a was measured by qRT-PCR, and U6 snRNA served as a control. **(H)** HPAC and PANC-1 cells were transfected with DGCR5 or si-DGCR5, respectively. The RNA level of miR-16 was measured by qRT-PCR, and U6 snRNA served as a control.

### DGCR5 inhibits chemoresistance of pancreatic cancer cells to 5-FU

To determine the possible role of DGCR5 in the response of pancreatic cancer cells to 5-FU, we generated 5-FU-resistant HPAC and PANC-1 cells. As shown in Figure 3A, compared with parental HPAC and PANC-1 cells, the IC50 of 5-FU was significantly increased in the 5-FU-resistant cells. The expression level of DGCR5 in 5-FU-resistant HPAC and PANC-1 cells was significantly reduced compared with that in parental cells (Figure 3B), which indicates that DGCR5 may be involved in the regulation of the response of pancreatic cancer cells to 5-FU response. To further investigate the role of DGCR5 in the response of HPAC and PANC-1 cells to 5-FU, we knocked down DGCR5 in parental and 5-FU-resistant HPAC cells and measured the IC50 of 5-FU in the cells. We found that the knockdown of endogenous DGCR5 increased the IC50 in both parental and 5-FU-resistant HPAC cells (Figure 3C); in contrast, the expression of exogenous DGCR5 reduced the IC50 of 5-FU in both parental PANC-1 and 5-FU-resistant PANC-1 cells (Figure 3D). Overall, these results imply that DGCR5 is involved in the regulation of the response of pancreatic cancer cells to 5-FU and in the reduction in chemoresistance of pancreatic cells to 5-FU.

### MiR-320a and DGCR5 mutually regulate each other in PDAC cells

LncRNAs exert various bio-functions. Some function as a scaffold, some are involved in the epigenetic modification of chromatin, and others regulate the function of microRNA. To determine whether DGCR5 functions as a regulator of microRNA, we conducted a microRNA array experiment to compare the discrepant expression of microRNA between PANC-1 cells transfected with the DGCR5 expression plasmid or the control plasmid. Several microRNAs were significantly differentially expressed between DGCR5-overexpressing cells and control cells (Figure 4A); miR-320a was one of most downregulated miroRNAs. We then used qRT-PCR to validate the results of the microarray (Figure 4B). Based on the results of the microarray and qRT-PCR experiments, we selected miR-320a for further study. A miR-320a target site was found in DGCR5 and was predicted by RegRNA (Figure 4C). We then constructed a luciferase reporter with a wild type DGCR5 fragment containing the miR-320a target site or its mutant form. MiR-320 expression was decreased, while anti-miR-320 increased the luciferase activity of the reporter gene with the WT fragment of DGCR5 (Figure 4D). Neither miR-320 nor anti-miR-320 exerted an influence on the reporter gene with the fragment of mutated DGCR5 (Figure 4E). We further measured the impacts of miR-320 on the expression level of DGCR5 by qRT-PCR. We found that miR-320 could reduce the level of DGCR5, while anti-miR-320 could increase the level of DGCR5 (Figure 4F). Together with the results of the reporter gene assay, we can conclude that miR-320 can directly regulate the level of DGCR5 in PDAC cells. Then, we found that overexpression of DGCR5 in HPAC cells reduced the miR-320 level, while the knockdown of DGCR5 in PANC-1 cells increased the level of miR-320a (Figure 4G). The impact of DGCR5 on miR-320 was specific, as the level of miR-16 was not affected by DGCR5 in either the PANC-1 or HPAC cell line (Figure 4H). Overall, these results demonstrate that miR-320a and DGCR5 mutually regulate each other in PDAC cells.

### DGCR5 reverses the inhibition of PDCD4 by miR-320a in PDAC cells

**Figure 5:**
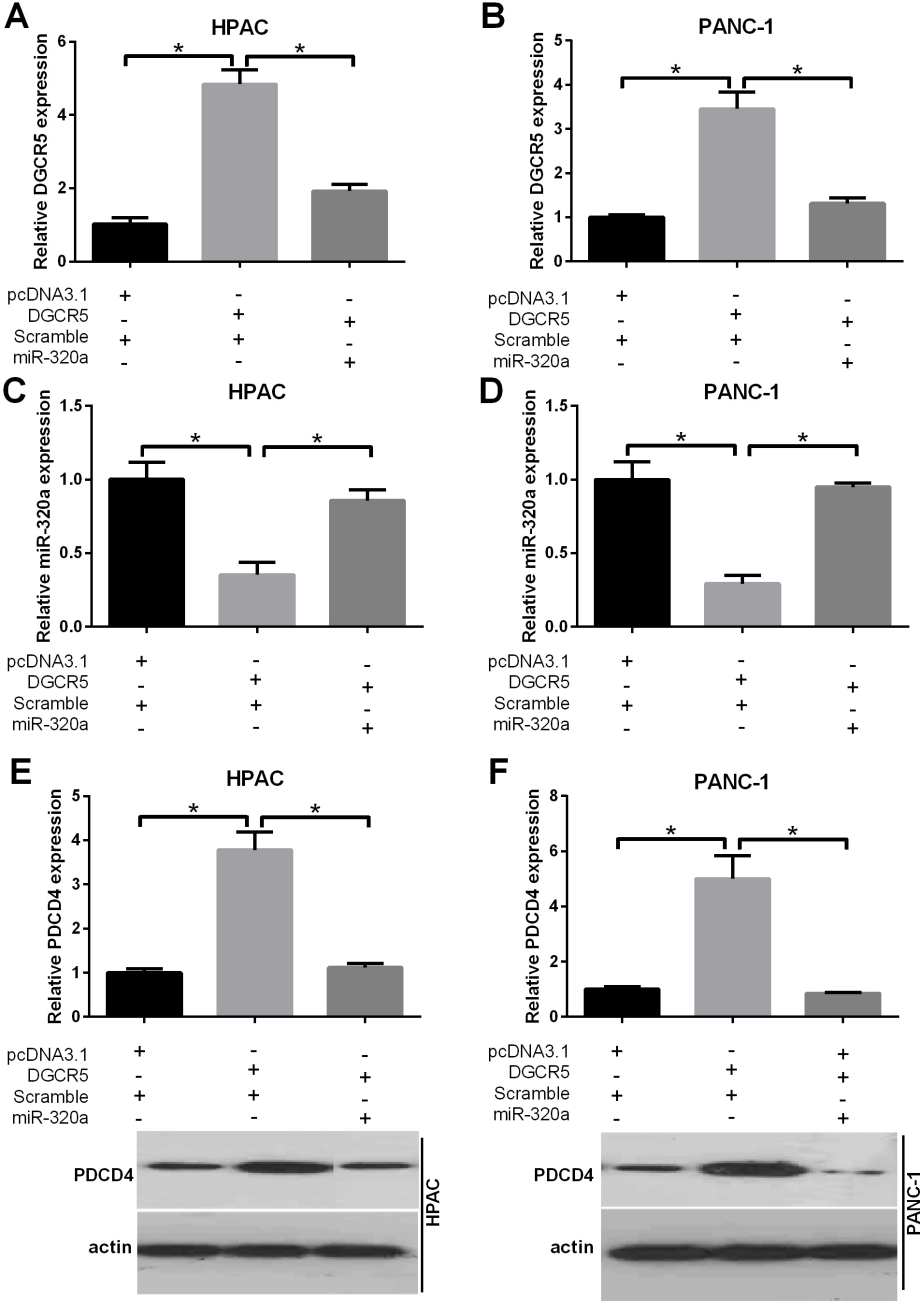
DGCR5 reverses the inhibition of PDCD4 by miR-320a (**A** and **B**) HPAC and PANC-1 cells were simultaneously transfected with DGCR5 and miR-320a mimics or control mimics, as indicated. The expression level of DGCR5 was measured by qRT-PCR, and GAPDH mRNA served as a control. (**C** and **D**) HPAC and PANC-1 cells were simultaneously transfected with miR-320a mimics and the DGCR5 expression plasmid or the control plasmid, as indicated. The expression level of miR-320a was measured by qRT-PCR, and U6 snRNA served as a control. (**E** and **F**) HPAC and PANC-1 cells were simultaneously transfected with DGCR5 and miR-320a mimics or control mimics, as indicated. The protein level of PDCD4 was measured by western blot, and GAPDH served as a loading control. The histogram represents data of three independent experiments. *p<0.05.

To further validate the mutual regulation of DGCR5 and miR-320a in PDAC cells, we performed a rescue assay. In both HPAC and PANC-1 cells, the DGCR5 expression plasmid significantly increased the level of DGCR5; the simultaneous transfection of these cells with miR-320a mimics reversed the efficiency of the DGCR5 expression plasmid (Figure 5A and 5B). Exogenous DGCR5 led to a reduction in miR-320a, while miR-320a mimics reversed the reduction in miR-320a (Figure 5C and 5D). Furthermore, we sought to determine whether DGCR5 affects the level of miR-320a target genes in PDAC cells. In transfected HPAC cells and PANC-1 cells, DGCR5 increased the protein level of PDCD4, while the simultaneous transfection of these cells with miR-320a mimics reversed the impacts of exogenous DGCR5 (Figure 5E and 5F). These results imply that DGCR5 can reverse the inhibition of PDCD4 by miR-320a in PDAC cells.

### MiR-320a mimics rescue the change in phenotype induced by DGCR5

**Figure 6:**
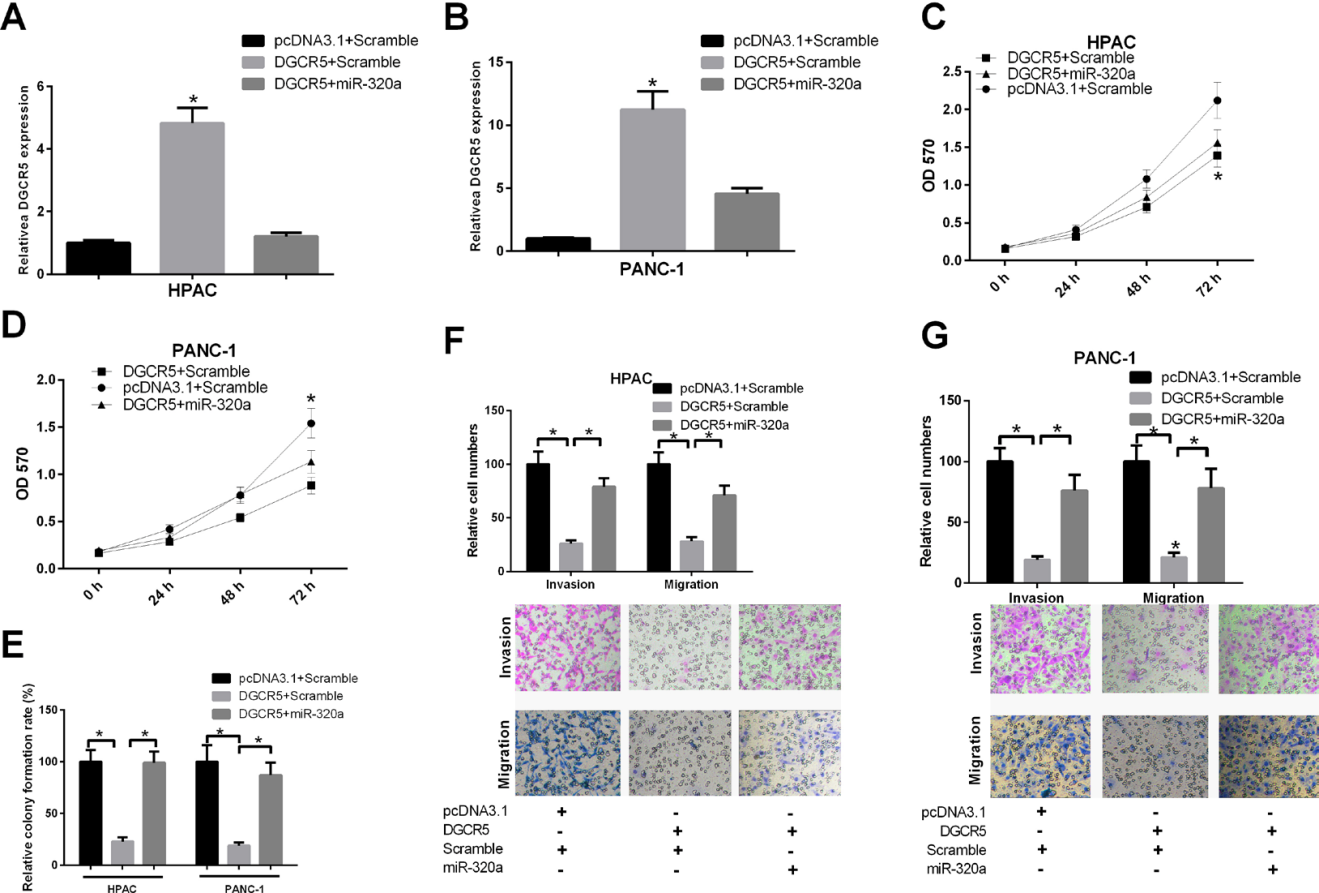
MiR-320a can reverse the impact of DGCR5 on the malignant phenotype of PDAC cells (**A** and **B**) HPAC and PANC-1 cells were simultaneously transfected with DGCR5 and miR-320a mimics or control mimics, as indicated. The expression level of DGCR5 was measured by qRT-PCR, and GAPDH mRNA served as a control. (**C** and **D**) HPAC and PANC-1 cells were simultaneously transfected with DGCR5 and miR-320a mimics or control mimics, as indicated. Cell proliferation was measured by CCK-8 assay. **(E)** HPAC and PANC-1 cells were simultaneously transfected with DGCR5 and miR-320a mimics or control mimics, as indicated. A colony formation assay was then performed. The histogram represents the results of three independent experiments. (**F** and **G**) The cells were transfected as mentioned above, and a Transwell migration and invasion assay was performed. The histogram represents the results of three independent experiments. *p<0.05.

To determine whether the impacts of DGCR5 on PDAC cell phenotype could be reversed by miR-320a mimics, we first transfected HPAC or PANC-1 cells with the DGCR5 expression plasmid alone or simultaneously with miR-320a; the qRT-PCR results demonstrated that the transfection was efficient (Figure 6A and 6B). A CCK-8 assay demonstrated that DGCR5 inhibited cell proliferation, while miR-320 mimics partially reversed the effect of DGCR5 (Figure 6C and 6D). The impact of DGCR5 on the colony formation ability of PDAC cells was also abrogated by miR-320a mimics (Figure 6E). We further conducted a Transwell migration and invasion assay to evaluate the role of miR-320a in the regulation of the phenotype of PDAC cells by DGCR5. The simultaneous transfection with miR-320a mimics abolished the effect of DGCR5 on the migration and invasiveness of HPAC and PANC-1 cells (Figure 6F and 6G). Together, these results demonstrate that DGCR5 modulates the phenotype of PDAC cells in a miR-320a-dependent manner.

### MiR-320a is involved in 5-FU resistance modulated by DGCR5

**Figure 7:**
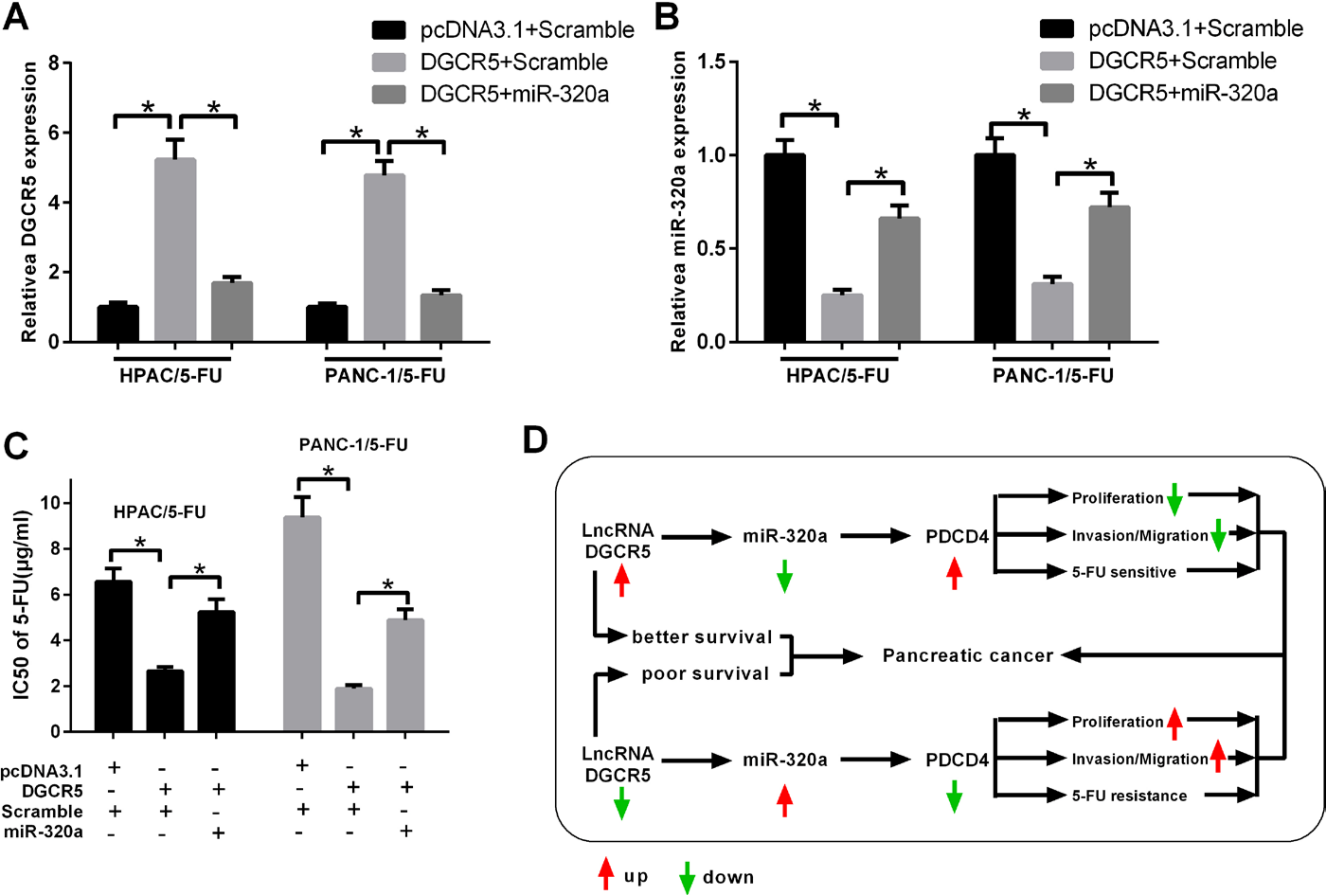
MiR-320a mediates the impact of DGCR5 on the resistance of PDAC cells to 5-FU **(A)** 5-FU-resistant HPAC and PANC-1 cells were transfected, as indicated. The expression level of DGCR5 was measured by qRT-PCR, and GAPDH mRNA served as a control. **(B)** 5-FU-resistant HPAC and PANC-1 cells were transfected, as indicated. The expression level of miR-320a was measured by qRT-PCR, and U6 snRNA served as a control. **(C)** 5-FU-resistant HPAC and PANC-1 cells were transfected, as indicated. The IC50 of 5-FU was measured by MTT assay. **(D)** A schematic model of lncRNA DGCR5 in PDAC.

We attempted to determine whether miR-320 is involved in the resistance of PDAC cells to 5-FU, which is regulated by DGCR5. We transfected 5-FU-resistant HPAC or PANC-1 cells with DGCR5 alone or simultaneously with miR-320a mimics, and the qRT-PCR results confirmed the transfection efficiency (Figure 7A and 7B). The IC50 of the cells was measured by MTT assay, and it was found that exogenous DGCR5 reduced the IC50 of both cell lines and that miR-320a mimics reversed the effect of DGCR5 (Figure 7C). This indicates that miR-320a is involved in the regulation of 5-FU resistance in HPAC and PANC-1 cells by DGCR5.

In this study, we demonstrated that the lncRNA DGCR5 was significantly reduced in both clinical PDAC samples and PDAC cell lines and that a lower DGCR5 level might imply a poor survival. In addition, it was found that miR-320a and DGCR5 mutually regulated each other and that DGCR5 reversed the inhibition of the miR-320a target gene PDCD4, which in turn inhibited the proliferation, migration and 5-FU resistance of PDAC cells (Figure 7D).

## DISCUSSION

An increasing amount of evidence has demonstrated that ncRNAs participate in the progression of many cancers including pancreatic cancer. The role of microRNAs in PDAC has been systematically studied, but as novel regulatory molecules, the role of lncRNAs in PDAC has not been well studied. Only a few studies have investigated the role of lncRNAs in PDAC, and considering the potential clinical and prognostic significance of lncRNAs in PDAC, more extensive studies in this field are required. In this study, we found that the lncRNA DGCR5 was significantly downregulated in PDAC tissues compared with non-PDAC pancreatic tissues. It was also revealed that DGCR5 is involved in the regulation of proliferation, migration and 5-FU resistance of PDAC cells. Further experiments suggested that DGCR5 and miR-320a mutually regulate each other and that DGCR5 reverses the inhibition of PDCD4 by miR-320a.

DGCR5 was first reported to be downregulated in Huntington’s disease [16], and other studies have demonstrated that DGCR5 plays a role in the progression of many cancers including lung cancer [17] and hepatocellular carcinoma [14]. DGCR5 was demonstrated to be a tumor suppressor gene in both lung cancer and HCC, and in accordance with this finding, our study found that DGCR5 could inhibit the proliferation and migration of PDAC cells. Further investigation demonstrated that DGCR5 promotes the sensitivity of PDAC cells to 5-FU, which indicates a tumor suppressive role for DGCR5 in PDAC. In previous studies, HOTAIR and PVT1 were demonstrated to be novel biomarkers for the early diagnosis of pancreatic cancer [5]. DGCR5 has been associated with the prognosis of both lung cancer and HCC [14, 17], and in our study, we found that a lower DGCR5 level correlated with a poor prognosis in patients with PDAC. The area under the ROC curve of DGCR5 indicated the potential diagnostic value of DGCR5 in PDAC.

Unlike protein coding genes, lncRNAs elicit their functions via various pathways. Some lncRNAs function as a scaffold on which many protein factors bind together to form a large functional unit [18–20], while other lncRNAs are involved in the regulation of chromatin epigenetic modification via the cis or trans method [20, 21]. Moreover, some other lncRNAs may function as sponges that regulate the activities of microRNAs [22, 23]. In our study, we found that DGCR5 could modulate the level of miR-320a, which has been demonstrated to promote the proliferation and migration of PDAC cells [24]. LncRNAs may contain many target sites for specific microRNAs and may compete with microRNA targets to bind to microRNA; this, in turn, reverses the inhibition of the target by that particular microRNA [22, 23]. We found that through the regulation of the level of miR-320a, DGCR5 could increase the level of the miR-320a target PDCD4, which has been shown to inhibit proliferation and to promote the sensitivity of PDAC cells to 5-FU [25, 26]. A miR-320a mimic reversed the impacts of DGCR5 on cell proliferation, migration and 5-FU sensitivity in PDAC cells, which further supports the functional relationship between these two molecules. Our data indicated that DGCR5 may be an endogenous sponge of miR-320 and that it can compete with miR-320a targets for binding to miR-320a, which would result in the upregulation of miR-320a target genes.

In conclusion, here we showed the way in which a novel lncRNA (DGCR5) regulates the progression and chemotherapeutic response of PDAC. DGCR5 and miR-320a reciprocally regulate each other, and DGCR5 reverses the inhibition of PDCD4 by miR-320a, which in turn affects the phenotype and chemotherapeutic response of PDAC cells. Downregulated DGCR5 in PDAC tissues indicates a poor prognosis for patients, and the area under the ROC curve suggested that DGCR5 might have potential diagnostic value for PDAC. Our study sheds new light on the molecular mechanism of PDAC progression and response to 5-FU with respect to lncRNA.

## MATERIALS AND METHODS

### Patient samples and cell lines

Thirty matched primary pancreatic cancer specimens and matched adjacent non-tumorous pancreatic tissues were collected from Huai’an First People’s Hospital of Nanjing Medical University with informed consent of the patients. The samples were placed into liquid nitrogen and then transferred to a freezer for storage at -80°C. All the procedures were in accordance with the guidelines of the ethics committee of the university. All cell lines were purchased from ATCC and were maintained according to the guidelines of ATCC.

### RNA extraction and qRT-PCR

Total RNA of the clinical samples and the cell lines was extracted with TRIzol reagent (Takara, Shiga, Japan) per the manufacturer’s instructions, and cDNAs were synthesized with a PrimeScript RT Reagent Kit (Perfect Real Time, Takara, Shiga, Japan) according to the standard protocol provided by the manufacturer. Real-Time PCR and SYBR Premix Ex Taq II (Takara, Japan) was conducted to detect the expression level of target genes. The level of DGCR5 was normalized to that of GAPDH, and the level of miR-320a was normalized to that of U6 snRNA; the fold change in the expression of the target genes was calculated using the 2 ^–ΔΔCt^ method.

### Cell proliferation and colony formation assay

The cells transfected with the DGCR5 expression plasmid, the knockdown plasmid or the respective control were seeded into a 96-well plate at an initial density of 4,000 cells per well. CCK8 was added to the culture medium at 24 h, 48 h, 72 h or 96 h after the cells were seeded. The absorbance at 450 nm was then detected using a microplate reader. For the colony formation assay, approximately 400 cells per well were seeded in a 24-well plate, and the medium was replaced every two days. Sixteen days after seeding, the cells were fixed and the number of colonies was counted.

### Transwell migration and invasion assay

The cells transfected with the DGCR5 expression plasmid, the knockdown plasmid or the respective control were seeded into a chamber coated with or without fresh Matrigel (diluted 1:6 in serum-free medium) (BD Biosciences San Jose, CA, USA) in a 24-well plate; approximately 2 × 10 ^5^ cells were seeded. The medium in the chamber did not contain serum, while the medium in the 24-well plates contained 20% FBS. Then, 36 hours after seeding, the cells that did not pass through the filter were removed by a cotton swab, whereas cells on the lower surface were fixed and stained with formaldehyde and crystal violet, respectively. The cells on the lower side of the filters were then counted.

### IC50 of 5-FU

The cells transfected with the DGCR5 expression plasmid, the knockdown plasmid or the respective control were seeded into a 96-well plate at an initial density of 4,000 cells per well. A different concentration of 5-FU was added to the medium, and each concentration was tested in triplicate. Twenty-four hours after 5-FU treatment, an MTT assay was conducted, and the well with 50% of 570 nm absorbance of the well without 5-FU treatment was the IC50 of 5-FU.

### Statistical analysis

Data are presented as the means ± SD. SPSS 22.0 software (IBM Corp., Armonk, NY, USA) was used for the statistical analysis. The survival calculations were illustrated with Kaplan-Meier curves, and differences between the survival curves were tested by the log-rank test.
